# Identification of genes and critical control proteins associated with inflammatory breast cancer using network controllability

**DOI:** 10.1371/journal.pone.0186353

**Published:** 2017-11-06

**Authors:** Ryouji Wakai, Masayuki Ishitsuka, Toshihiko Kishimoto, Tomoshiro Ochiai, Jose C. Nacher

**Affiliations:** 1 Department of Information Science, Faculty of Science, Toho University, Miyama 2-2-1, Funabashi, Chiba 274-8510, Japan; 2 Department of Molecular Biology, Faculty of Science, Toho University, Miyama 2-2-1, Funabashi, Chiba 274-8510, Japan; 3 Proteome Analysis Center, Faculty of Science, Toho University, Miyama 2-2-1, Funabashi, Chiba 274-8510, Japan; 4 Faculty of Social Information Studies, Otsuma Women’s University, 2-7-1 Karakida, Tama-shi, Tokyo 206-8540, Japan; Tokyo Institute of Technology, JAPAN

## Abstract

One of the most aggressive forms of breast cancer is inflammatory breast cancer (IBC), whose lack of tumour mass also makes a prompt diagnosis difficult. Moreover, genomic differences between common breast cancers and IBC have not been completely assessed, thus substantially limiting the identification of biomarkers unique to IBC. Here, we developed a novel statistical analysis of gene expression profiles corresponding to microdissected IBC, non-IBC (nIBC) and normal samples that enabled us to identify a set of genes significantly associated with a specific disease state. Second, by using advanced methods based on controllability network theory, we identified a set of critical control proteins that uniquely and structurally control the entire proteome. By mapping high change variance genes in protein interaction networks, we found that a large statistically significant fraction of genes whose variance changed significantly between normal and IBC and nIBC disease states were among the set of critical control proteins. Moreover, this analysis identified the overlapping genes with the highest statistical significance; these genes may assist in developing future biomarkers and determining drug targets to disrupt the molecular pathways driving carcinogenesis in IBC.

## Introduction

The increasing availability of high-quality transcriptomic data for specific diseases offers the possibility of uncovering complex statistical patterns hidden in gene expression profiles. The discovered patterns may eventually be combined with the collective knowledge conferred by the newly assembled large-scale interactome, thus offering a novel and promising framework to investigate complex biological phenomena as well as the roots of genetic diseases [1–6]. Recent research has emphasized that a genetic perturbation of a single gene product is not usually responsible for the emergence of a disease phenotype [2]. In contrast, the complex cellular network built through multiple physical bindings and chemical reactions among molecules contains pathological processes that lead to complex disorders. This information suggests that the network can be potentially disrupted in distant locations whose perturbation affects entire signalling pathways and molecular complexes, thus leading to the emergence of disease pathways and modules [1, 2].

The development of controllability methods to assess critical control as well as potential dysregulation locations in complex biological pathways and networks is an important topic in computational biology [1]. Maximum matching (MM) has recently been proposed as a novel method to identify driver nodes (i.e., controllers) in directed networks [7, 8]. However, some biological networks are undirected. More importantly, MM identifies a large number of potential controllers, especially in scale-free networks, a typical structure that is abundant in biological systems. In contrast, by using the minimum dominating set (MDS) approach, controllability methods can be applied to both undirected and directed networks [9–11] and bipartite networks [12, 13]. In addition, the set of driver nodes tends to be smaller, particularly in scale-free networks [9, 14]. Although the problem is NP-hard and it is not plausible that there exists an algorithm that can compute an MDS in polynomial time, pre-processing heuristics and fast algorithms have recently been proposed that not only drastically decrease the computational time (by more than 100 times) but also expand the solvable network size significantly [15]. This result paves the way to the application of MDS methods to investigate controllability in proteome-wide protein interaction networks [16]. The MDS method is increasingly being used for analysing biological networks from protein interaction networks, gene expression profiles, and metabolic networks to assessment correlation between cancer and control features and topological modules [11, 15, 17–20].

In particular, Wuchty found that in protein interaction networks the MDS tends to be enriched by cancer-related and virus-targeted genes [17]. Centrality-based MDS was also used to identify associations between essential and disease-related genes [19]. However, the MDS computation does not provide a unique solution, and several minimum dominating subsets may control the network. Critical nodes, in contrast, are unique and present in all of the MDS solutions. The development of critical controllability algorithms has led to several findings such as unveiling associations between critical control proteins and essential genes using integration of gene expression profiles with proteome-wide protein interaction networks [15]. Non-coding RNAs with critical network control roles were also associated to specific human diseases using bipartite networks [13]. See also the following review for details [11].

One of the most aggressive forms of breast cancer is inflammatory breast cancer (IBC), which occurs when the lymphatic vessels in breast skin are blocked by cancer cells. The absence of an evident tumour mass and its rapid development invading breast mass make a fast and accurate diagnosis crucial [21, 22]. Moreover, genomic differences between common breast cancers and IBC have not been completely assessed, thus greatly limiting the identification of biomarkers unique to IBC and the development of drugs for disrupting IBC associated molecular pathways.

Recent gene expression profiles analyses of IBC have also led to identify specific correlations such the response to neoadjuvant chemotherapy and metastasis-free survival [23]. However, critical controllability analysis of IBC gene expression profiles integrating large-scale protein interaction network has not been investigated yet, which is the focus of this research.

In this work, we developed a novel statistical analysis of gene expression profiles corresponding to microdissected IBC, non-IBC (nIBC) and normal samples [21]. This statistical analysis enabled us to identify specific genes that are significantly associated with a specific disease. Second, by using advanced methods based on controllability theory, we identified a set of critical control proteins that are located in the human protein-protein interaction network and can uniquely and structurally control the entire proteome. We then mapped the statistically identified genes associated with a disease state onto a protein interaction network and evaluated the degree of overlap between these genes and the identified set of critical control proteins. The results suggested that a large statistically significant fraction of the genes associated with IBC and nIBC diseases were also among the set of critical control proteins. Moreover, this analysis identified the overlapping genes with the highest statistical significance; these genes may assist in eludicating specific IBC associated molecular pathways as well as in future drug developments.

## Collected data

The microarray gene expression data for IBC were based on frozen tissue samples obtained from core biopsies analysed by the University of Texas. Microdissection of tissue produces high-quality gene expression data and significant noise reduction [21]. In addition, the full dataset included nIBC and normal data and was downloaded from the publicly available GEO database. In this analysis, the dataset consisted of genome-wide gene expression profiles with 20 samples of IBC, 20 of nIBC and five of normal state. Each sample corresponded to a patient and included 40,990 gene probes. A preliminary analysis was conducted to determine the genes among the replicated probes before the genes were mapped to the human protein interaction network. The *H. sapiens* protein interaction network was compiled from HINT Database High-quality Interactomes Version 3 [16] and consisted of 11,762 proteins (nodes) and 49,855 interactions (edges). (See Supporting Information S1 File.)

## Methods

### Average and variance changes among three states

Let Ω be the collection of all probes. Let $x_{i}^{A}\left(t\right)$ be the gene expression level, where *t* is the number of data points (samples), *i* ∈ Ω denotes a probe, and *A* indicates one of three states: nIBC, IBC, and normal. In other words, $x_{i}^{nIBC}\left(t\right)$, $x_{i}^{IBC}\left(t\right)$, and $x_{i}^{normal}\left(t\right)$ are the gene expression data for nIBC, IBC and normal, respectively. Then, the average and variance for state A is given by

$$\begin{array}{c} {\bar{x}}_{i}^{A}=\frac{1}{N}\sum_{t}^{N}x_{i}^{A}\left(t\right), \end{array}$$ (1)

$$\begin{array}{c} v_{i}^{A}=\frac{1}{N-1}\sum_{t}^{N}{\left(x_{i}^{A}\left(t\right)-{\bar{x}}_{i}^{A}\left(t\right)\right)}^{2}, \end{array}$$ (2)

where *N* is the number of data points (samples).

To examine whether the average is changed from the normal state to the disease state, we define the change ratio of the averages between A and B state as follows:

$$\begin{array}{c} c_{i}^{\left(A,B\right)}=\log\left({\bar{x}}_{i}^{A}/{\bar{x}}_{i}^{B}\right). \end{array}$$ (3)

Similarly, to examine whether the variance is changed from the normal state to the disease state, the change ratio of the variances between state A and state B is given by:

$$\begin{array}{c} d_{i}^{\left(A,B\right)}=\log\left(v_{i}^{A}/v_{i}^{B}\right). \end{array}$$ (4)

**Fig 1:**
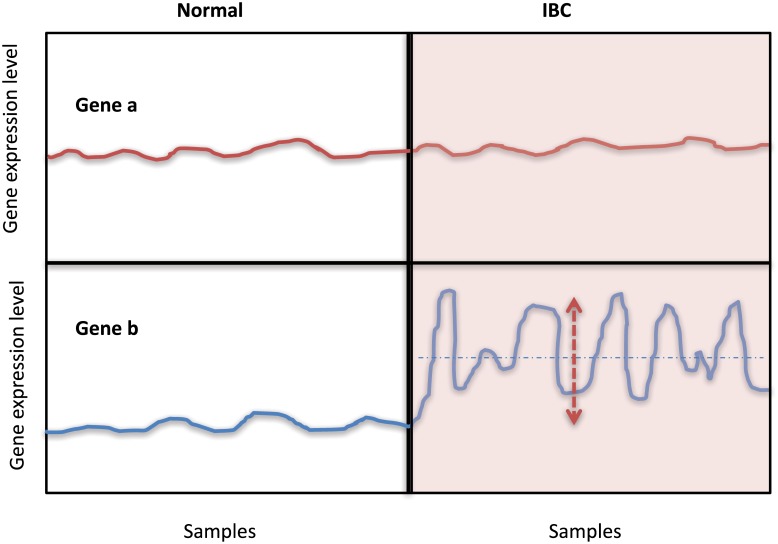
Illustration of the statistical concept observed in our analysis. We observed two representative samples (gene a and gene b) of expression dynamics when transitioning from two different states. First, the gene a does not show significant changes in both average and variance in both states. However, the gene b exhibits high change of both average and variance when transitioning from normal to IBC state.

Here, the possible pairs are (*A*, *B*) = (*nIBC*, *normal*), (*IBC*, *normal*) and (*IBC*, *nIBC*). This expression measures the differential variability of the expression of each gene between two states (see Fig 1). In other words, it enables selection of genes whose variance changes significantly from state A to state B (for example, normal state (A = normal) to disease state (B = nIBC or IBC). As we will discuss in the next section, we mainly used $d_{i}^{\left(A,B\right)}$ rather than $c_{i}^{\left(A,B\right)}$ to check the association of dynamic change nodes and critical nodes.

To select the genes (probes) whose variance in the disease state was significantly changed from that of the normal state, we use a one-tailed F-test. We compute the p-value with a null hypothesis such that the population variance of state A ($v_{i}^{A}$) is equal to that of state B ($v_{i}^{B}$). The ratio of the sample variance follows an F-distribution under the null hypothesis. Then, we can compute the p-value for the observed $v_{i}^{A}/v_{i}^{B}$ as follows:

$$\begin{array}{c} p_{i}^{\left(A,B\right)}=P\left(F>v_{i}^{A}/v_{i}^{B}\right), \end{array}$$ (5)

where *F* is a random variable from the F-distribution with parameters *N*_1_ and *N*_2_. Here, *N*_1_ and *N*_2_ are the number of data points (samples) of states A and B, respectively. A small p-value suggests that for that probe (gene), the variance in the disease state is significantly different from that in the normal state.

The original dataset contained several probes for each gene. Therefore, we statistically select one probe as a representative for each gene to map the gene products to the protein-protein interaction network. We then select from multiple probes one probe that corresponds to the same gene by selecting the probe with the lowest p-value. Let Ω_0_ (Ω_0_ ⊂ Ω) be the collection of all filtered probes that uniquely correspond to only one gene. Then, by identifying each gene by the corresponding lowest p-value probe, we consider that Ω_0_ is the set of genes to be mapped to the protein-protein interaction network.

### Controllability in protein interaction networks

Recently, several structural controllability methodologies have been proposed to investigate associations between controllers (i.e., driver nodes) and biological functions. Among them, the MDS approach has been successfully applied to biological networks to determine associations between life molecules and biological functions [11]. Through the MDS method, statistical associations between the sets of cancer genes and virus target genes and the set of structural controllers have been uncovered [17]. However, the MDS method does not provide a unique solution, and several subsets of nodes may potentially control the entire network. To uniquely identify the subset of nodes that control the whole network, the nodes must be categorized into critical, intermittent and redundant nodes [10]. Those nodes belonging to the critical set are, therefore, present in all of the MDS solutions. This set of nodes is thought to be the most important in terms of controllability features. Because the MDS problem is NP-hard, it is not plausible that there exists an algorithm that can compute the solution in polynomial time. Therefore, it is very difficult to solve the critical MDS problem in a large-scale network, such as the human protein-protein interaction network. However, recent developments have led to the discovery of a new algorithm that uses a preprocessing step that not only significantly decreases the computational time but also expands the computable network size [15]. In this work, we used and applied this algorithm to identify the critical set of nodes present in a proteome-wide protein-protein interaction network by using the HINT database.

To identify the MDS and the critical set, we formalized a problem that can be solved using Integer Linear Programming (ILP) [9]. The MDS problem can be formalized as an ILP problem as follows:

minimize

$$\begin{array}{c} \sum_{v\in V}x_{v}, \end{array}$$ (6)

subject to

$$\begin{array}{c} x_{v}+\sum_{(u,v)\in E}x_{u}\geq 1\;\;\;\left(\forall v\in V\right), \end{array}$$ (7)

$$\begin{array}{c} x_{v}\in \left\{0,1\right\}\;\;\;\left(\forall v\in V\right). \end{array}$$ (8)

The critical set should consist of nodes that are present in all of the possible solutions of an MDS. Therefore, the ILP should be solved many times, thus slowing the computation. To address this drawback, new methods have been developed by Ishitsuka et al that apply some pre-processing steps that simplify the resulting ILP problem and speed up the computation [15]. The algorithmic steps are as follows:

First, we apply the critical proposition for each node, which states that if node *v* has two or more neighbouring nodes with degree *k* = 1, *v* is a critical node. Second, we apply the redundant proposition for each remaining node, which states that if all neighbours of a node *v* are critical nodes, *v* is a redundant node [15].

After these two preprocessing steps have been considered, we then proceed as in [10] to determine all control categories of all nodes. Because these preprocessing heuristics largely simplified the MDS problem, it is possible to speed up the computation of control categories and obtain the solution even in large neworks [15].

### Determining associations between high change variance nodes and critical nodes

In this work, we examine whether the critical nodes are statistically associated with a high change in variance nodes. Recently, methods based on fluctuation dynamics, such as volatility-constrained correlation, have been applied to predict control directionality in gene regulation using gene expression profiles [24]. Here, we first provide a general method to determine whether a subset of nodes is statistically associated with a high change variance nodes. Furthermore, we use a two-tail binomial test to estimate the statistical significance of this association.

To determine genes with high change in variance, we select genes below the threshold *χ* for p-value $p_{i}^{\left(A,B\right)}$ defined in the previous section. We define the group of the selected genes *α*^(*A*,*B*)^ and the remaining set of genes *β*^(*A*,*B*)^ as follows:

$$\begin{array}{ccc} \alpha^{\left(A,B\right)} & = & \left\{i\in \Omega_{0}:p_{i}^{\left(A,B\right)}<\chi \right\} \end{array}$$ (9)

$$\begin{array}{ccc} \beta^{\left(A,B\right)} & = & \{i\in \Omega_{0}:p_{i}^{\left(A,B\right)}>\chi \}. \end{array}$$ (10)

In other words, *α*^(*A*,*B*)^ is the group of higher change variance genes, and *β*^(*A*,*B*)^ is the group of lower change variance genes. Then, the probability that one gene belongs to *α*^(*A*,*B*)^-group is given by

$$\begin{array}{c} r=\frac{\#\alpha^{\left(A,B\right)}}{\#\alpha^{\left(A,B\right)}+\#\beta^{\left(A,B\right)}}, \end{array}$$ (11)

where #*α*^(*A*,*B*)^ and #*β*^(*A*,*B*)^ are the number of the elements of *α*^(*A*,*B*)^ and *β*^(*A*,*B*)^, respectively. In other words, *r* is the probability that the variance of one genes changes significantly between the normal and the disease state, and significance is determined by threshold *χ*.

To identify a statistically significant association between the set of high change variance nodes and the set of critical nodes, we perform the following calculations: Let us consider a subset of genes Σ ⊂ Ω_0_. Let *η* be the number of Σ. Then, *rη* is the expected number of Σ in *α*-group. However, if we find that the actual number of Σ in *α* group is more than the expected value *rη* with high statistical significance, then we assume that Σ is related to higher change variance genes. Statistical significance is computed by a two-tail binomial test. Note that the p-value of this test is given by

$$\begin{array}{c} \tilde{p}=2\times P\left(X>x\right) \end{array}$$ (12)

where *X* is the random variable of the binomial distribution with probability *r* and total number *η*, and *x* is observed number of critical nodes in alpha groups. If *x* < *rη*, we compute the p-value by taking the opposite tail.

## Results and discussion

### Probability density shift towards high change ratio of gene expression in nIBC and IBC from normal state

**Fig 2:**
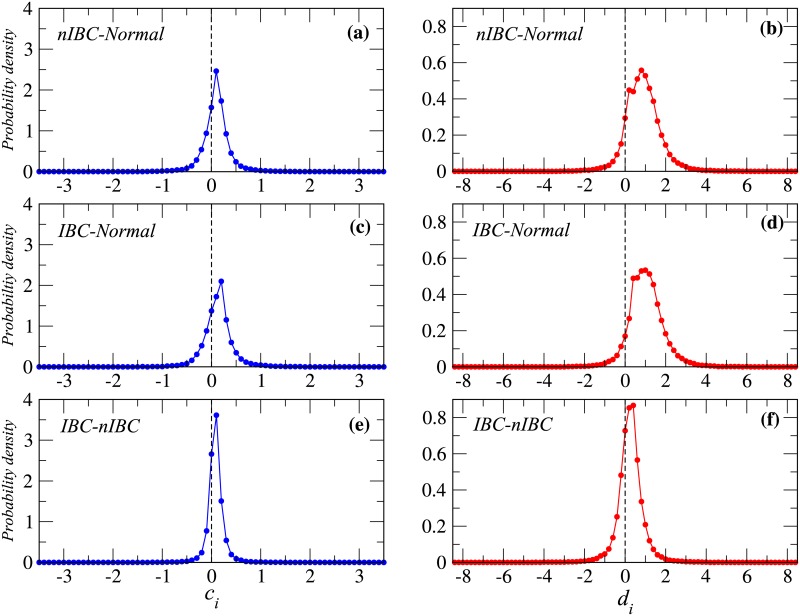
The probability density for the *c*_*i*_ (a,c,e) and *d*_*i*_ (b,d,f) change ratios for all three state pairs. The results clearly show a displacement of the distribution to the right hand size. This shift is more significant in the case of *d*_*i*_ change ratio. The disease state pairs are indicated in figure.

**Fig 3:**
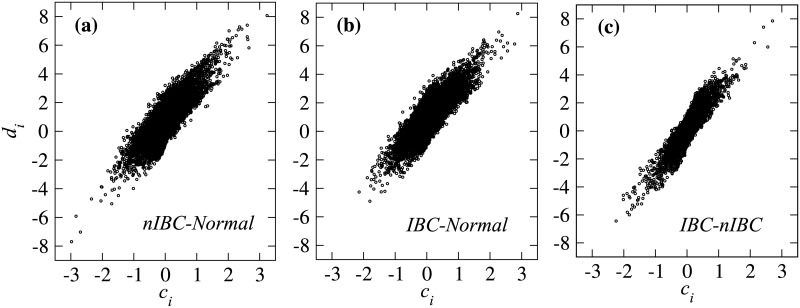
Comparison of the distribution (*c*_*i*_, *d*_*i*_) for all three state pairs. The distribution of (*c*_*i*_, *d*_*i*_) for nIBC-normal, IBC-normal, IBC-nIBC state pairs, respectively. The results show that *c*_*i*_ and *d*_*i*_ are linearly scaled.

In Fig 2(a)–2(f), we show $c_{i}^{\left(A,B\right)}$ and $d_{i}^{\left(A,B\right)}$ for the three state pairs (*A*, *B*) = (*nIBC*, *normal*), (*IBC*, *normal*), and (*IBC*, *nIBC*), respectively. The results show that disease states have a higher average than those of normal states (Fig 2(a) and 2(c)). Moreover, the disease states also have a larger variance than that of normal states (Fig 2(b) and 2(d). Although these observed signals are weaker for the (*IBC*, *nIBC*) states, the shift is also present in both average and variance distributions (Fig 2(e) and 2(f)), thus showing that the IBC state induces the largest differential change in gene expression. Furthermore, we also see that signal of *d*_*i*_ (change in variance) is more significant than that of *c*_*i*_ (change in average) (see Fig 2(a), 2(b), 2(c) and 2(d)). The coefficient of variation (CV) is defined as the ratio of the standard deviation (STD) to the average. Table 1 shows that the results for the CV of *d*_*i*_ are lower than those of *c*_*i*_ in (*A*, *B*) = (*nIBC*, *normal*) and (*IBC*, *normal*) cases. This result suggests that *d*_*i*_ (change in variance) is a better indicator than *c*_*i*_ for discriminating a disease state from a normal state. Therefore, in the next section, we focus on the change in variance *d*_*i*_ rather than *c*_*i*_ (change in average). Next, in Fig 3(a)–3(c), we show the distribution of (*c*_*i*_, *d*_*i*_) for nIBC-normal, IBC-normal, and IBC-nIBC state pairs, respectively. These results indicate that *c*_*i*_ and *d*_*i*_ are linearly scaled.

**Table 1:** Statistical results for the average, variance and CV corresponding to the data shown in Fig 2.

Pair	nIBC-normal	IBC-normal	IBC-nIBC
ave of *c*_*i*_	0.050938	0.08245	0.03151
std of *c*_*i*_	0.284793944	0.2989	0.180037
CV of *c*_*i*_	5.5909	3.6263	5.7133
ave of *d*_*i*_	0.77605	0.9355	0.15944
std of *d*_*i*_	0.86325	0.8554	0.6559
CV of *d*_*i*_	1.1123	0.91447	4.11362

### Proteins engaged in structural critical control also exhibit a high change in expression variance

Previous analyses have shown the importance of identifying the minimum driver set (MDS) of nodes required to control a complex network. The methods that determine the MDS have been applied to several real biological networks and systems. By using newly developed methods that allowed us to compute the MDS in large networks, we investigated the association between structural critical control nodes and high change in variance nodes in terms of gene expression level in transitioning from normal to disease states. First, we computed (1) the critical control set of proteins by applying an algorithm from Ishitsuka et al to a large-scale human protein-protein interaction network [15] The results showed that 10% of proteins were classified as critical nodes. The redundant set of proteins represented 60% of the entire proteome. The intermittent set of proteins, which control some network configurations, represented 30% of all proteins. The critical set of proteins was the smallest fraction and was used in our analysis to examine the association between dynamic gene expression features and structural controllability. Second, we mapped (2) the set of genes exhibiting a high change in expression variance on this network and calculated the statistical association between both sets of nodes (1) and (2).

Note that because the change in variance was more significant than the change in the average, as described in the previous section, we used $d_{i}^{\left(A,B\right)}$ rather than $c_{i}^{\left(A,B\right)}$, thus uncovering an association between critical nodes and nodes with high changes in variance.

Let *C* be the set of critical nodes. Let *η* be the number of overlaps Σ = *C* ∩ Ω_0_, which are critical nodes overlapping with the mapped genes from expression data. We compute the probability *r* that one gene belongs to the high change variance nodes group *α*^(*A*,*B*)^ by Eq (9).

If critical nodes are not related to high change variance nodes, *rη* is the expected number of critical nodes in *α*^(*A*,*B*)^-group. However, we find that the actual number of critical nodes *x* in *α*^(*A*,*B*)^ group is more than the expected value *rη* with high statistical significance.

The computation results are shown in Table 2. As an example, we explain in detail the case for nIBC-normal. We consider two thresholds, *χ* = 0.05 and 0.01, for grouping high variance change set of genes *α*^(*nIBC*,*normal*)^. For the threshold *χ* = 0.05, the probability that one gene belongs to high change variance group *α*^(*nIBC*,*normal*)^ is *r* = 0.5158. Thus, the expected number of critical node in *α*^(*nIBC*,*normal*)^ is *rη* = 336.86. However, the actual number of critical nodes in *α*^(*nIBC*,*normal*)^ (*x* = 441) is much larger that the expected value, with statistical significance (p-value: $\tilde{p}=2.2\times 10^{-16}$). In similar way, nIBC-normal (*χ* = 0.01) and IBC-normal (*χ* = 0.05 and 0.01) cases exhibit similar tendancies (i.e., critical nodes are present in high variance change group more than expected with high statisitcal significance). This relationship is, however, not observed for the IBC-nIBC pair.

**Table 2:** Results of the statistical analysis for the association between critical proteins and genes with a high change in variance for three disease state pairs. *η* = 653.

pair	threshold *χ*	*r*	expected (r*η*)	actual (x)	p-value ($\tilde{p}$)
nIBC-normal	0.05	0.5158	336.86	441	2.2 × 10^−16^
nIBC-normal	0.01	0.3210	209.64	282	2.50 × 10^−9^
IBC-normal	0.05	0.60211	393.18	456	2.61 × 10^−7^
IBC-normal	0.01	0.40299	263.15	326	5.53 × 10^−7^
IBC-nIBC	0.05	0.14128	92.260	79	0.147
IBC-nIBC	0.01	0.05852	38.21	36	0.792

This finding suggested that the critical gene products (proteins) are significantly associated with the high change variance genes in transitioning from normal state to disease state (either nIBC or IBC). In other words, the set of proteins engaged in structural critical control has a higher chance of having a high change in variance in gene expression level in transitioning to a disease state (IBC or nIBC) from a normal state. Table 3 shows the set of identified critical control genes with the largest change in variance in gene expression levels.

**Table 3:** Identified critical control genes with the largest change in variance in expression levels in transitioning from normal to IBC and nIBC disease states.

rank	nIBC-normal	IBC-normal
1	MAGEA12	CGA
2	FOXJ1	ACTN2
3	ACTN2	MAGEA12
4	CDC6	KRT31
5	KRT31	FOXJ1
6	NDUFA4L2	ERBB2
7	SPERT	SH3GL2
8	ERBB2	CDC6
9	GFI1B	ELN
10	MLX	GAL

### Identified critical genes and their functionality

The identified critical control genes ranked on the basis of the largest change in variance (smallest p-value) in gene expression levels in transitioning from normal to nIBC and IBC disease states are shown in Table 3. Among all the genes, only four were uniquely associated with the IBC state. Moreover, six genes were identified in both the nIBC and IBC disease states. Here, we investigated the functionality of these genes in the literature and whether they have been associated with cancer in previous studies (see Table 4).

**Table 4:** Annotation and functionality for the unique proteins identified in transitioning from normal to both IBC and nIBC states. Main GO Molecular functions are described in Table. GO Biological processes are discussed in main text.

Gene Symbol	Gene ID	UniProt	GO Molecular Function
CGA	1081	P01215	Hormone activity
ELN	2006	P15502	Extracellular matrix structural constituent
GAL	51083	P22466	Galanin receptor activity
MAGEA12	4111	P43365	Not known, though may play a role tumor transformation or progression
FOXJ1	2302	Q92949	DNA binding
ACTN2	88	P35609	Calcium ion binding
CDC6	990	Q99741	Nucleotide binding
KRT31	3881	Q15323	Structural constituent of cytoskeleton
ERBB2	2064	P04626	ATP binding
SH3GL2	6456	Q99962	Lipid binding

First, we considered the overlapping set of genes. MAGEA12 (MAGE family member A12), also known as melanoma-associated antigen 12 or cancer/testis family 1, member 12, has been reported to be highly expressed in multiple tumours and cancers, such as head and neck squamous cell carcinoma, lung and breast cancers and melanoma. This gene has been observed to be expressed in only normal testis tissues but not in other normal tissues [25–27]. This observation is consistent with our findings, because our results suggested strong associations in both IBC and other types of breast cancer (nIBC). Regarding gene ontology, the biological process associated with this gene is still unknown.

FOXJ1 (forkhead box J1) has been previously associated with allergic rhinitis (ALRH) disease susceptibility. This common disease caused by allergen exposure induces mucosal inflammation. Interestingly, variations in FOXJ1 expression levels are correlated with progression and tumour cell proliferation of gastric cancer and hepatocellular carcinoma [28, 29]. The related ontological biological processes include actin cytoskeleton organization, activation of GTPase activity, and brain and epithelium development. ACTN2 (actinin alpha 2) is a multi-role actin-binding protein in a large variety of cell types. The associated biological processes include MAPK cascade, actin filament uncapping, cardiac muscle cell, cell adhesion and negative regulation of potassium ion transmembrane transporter activity [30]. CDC6 (cell division cycle 6) is a gene that encodes a key protein with regulatory roles in the early steps of DNA replication. CDC6 protein is localized in the cell nucleus during cell cycle G1. However, when the S phase starts, CDC6 translocates to the cytoplasm. High expression of CDC6 is also correlated with accelerated cell proliferation in epithelial ovarian cancer [31, 32]. The associated biological processes include DNA replication, G1/S transition of the mitotic cell cycle, cell division and positive regulation of cytokinesis. KRT31 (keratin 31) encodes an acidic protein responsible for forming hair and nails [33]. The biological processes associated with this protein include cornification, cytoskeleton organization, epidermis development and keratinization. ERBB2 (erb-b2 receptor tyrosine kinase 2), also known as HER2, encodes a protein belonging to the epidermal growth factor (EGF) family of receptor tyrosine kinases. The gene ontology information indicates that HER2 participates in processes including the ERBB2 signalling pathway, MAPK cascade, cell proliferation and negative regulation of ERBB signalling pathway. The involvement of ERBB2 has been demonstrated in multiple human disorders. Indeed, it has been found to be overexpressed in approximately 30% of human breast cancers [34] and in many other cancers, including ovarian, stomach, bladder, salivary, and lung cancers [35]. There is strong evidence supporting a dysregulatory role of ERBB2 for normal cell-control mechanisms, thus leading to an aggressive form of tumour cells [36].

Among the highly ranked genes shown in Table 3, four were uniquely assigned to the transition between normal state to IBC disease. Here, we describe the biological functionality of these genes.

CGA (glycoprotein hormones, alpha polypeptide), also known as CHGA, encodes an alpha subunit protein and belongs to the glycoprotein hormones alpha chain family. Although little is known about CGA involvement in carcinogenesis, some research has found associations among CGA gene overexpression, gastric cancer occurrence and gastric cancer cell apoptosis. Mutations and abnormal protein expression of this gene have been found in several other cancers, such as lung, pancreatic, neuroblastoma, prostate cancers and pituitary tumours [37, 38]. The biological processes involved include cell-cell signalling, peptide hormone processing, positive regulation of cell migration, and positive regulation of cell proliferation. The ELN (elastin) gene is located in the Williams-Beuren syndrome (WBS) region. Haploinsufficiency of ELN has been suggested as the origin of the cardiovascular and musculoskeletal abnormalities observed in the disease. Researchers have found evidence of severe changes and even fragmentation of elastin in invasive type of tumours, in which those fibres are disrupted [39, 40]. The biological processes involved include animal organ morphogenesis, blood circulation, cell proliferation, extracellular matrix disassembly and extracellular matrix organization. The GAL (GAL galanin and GMAP prepropeptide) gene is widely expressed in a variety of human systems ranging from gastrointestinal, pancreas and urogenital tracts to central and peripheral nervous systems. More recently, higher expression of GAL has been associated with tumour recurrence among colorectal cancer patients [41]. Its related biological processes include cAMP-mediated signalling, feeding behaviour, inflammatory response, insulin secretion, and negative regulation of lymphocyte proliferation. Finally, SH3GL2 (SH3 domain containing GRB2-like 2), also known as Endophilin-A1, is frequently deleted in non-small cell lung cancer. This protein has been shown to downregulate tumour growth by modulating EGFR signalling [42]. The loss of Sh3gl2 is associated with increased tumour grade and with muscle invasion, which is a reliable predictor of metastatic disease and cancer-derived mortality [43]. The biological processes associated with this gene are antigen processing and presentation of exogenous peptide antigen via MHC class II, central nervous system development, membrane organization, microtubule-based movement, and negative regulation of the EGF receptor signalling pathway.

## Conclusion

The combination of structural controllability methodologies with high-quality gene expression profiles of breast cancer is a novel research direction that warrants further investigation. In this work, we found two main results. First, we unveiled a statistical feature hidden in the gene expression profiles of IBC and nIBC datasets. This feature showed that a subset of genes exhibit a high change in variance in the disease state compared with the normal state. These genes are therefore expected to play a key role in the transition from the normal state to both IBC and nIBC states. Second, by using a recently proposed algorithm for critical controllability, we were able to identify an optimal critical set of proteins that structurally controls the entire protein-wide protein interaction network. Our analysis established a statistical relationship between the set of critical proteins and the set of genes whose fluctuations are highly coupled to both breast cancer and IBC disease states.

Furthermore, the analysis of the identified critical control genes with the largest change in variance revealed that more than half of genes were responsible for both nIBC and IBC disease states. The genes uniquely associated with IBC were also examined in detail, and we found, through using different analytical methodologies, that a fraction of them were also associated with IBC disease, thus validating our results.

We believe that the presented results may encourage further research on both theoretical controllability analysis and biological experiments seeking to identifying drug targets to disrupt the molecular pathways driving carcinogenesis in IBC.

## Supporting information

S1 File The protein-protein interaction network data. This file contains the background data to construct the protein-protein interaction network.
(TXT)
